# Cannibalism and Necrophagy Promote a Resource Loop and Benefit Larval Development in Insects of Temporary Waters

**DOI:** 10.3390/insects12070657

**Published:** 2021-07-20

**Authors:** Valentina Mastrantonio, Graziano Crasta, Sandra Urbanelli, Daniele Porretta

**Affiliations:** 1 Department of Environmental Biology, Sapienza University of Rome, 00185 Roma, Italy; valentina.mastrantonio@uniroma1.it (V.M.); sandra.urbanelli@uniroma1.it (S.U.); 2 Department of Mathematics, Sapienza University of Rome, 00185 Roma, Italy; graziano.crasta@uniroma1.it

**Keywords:** ephemeral habitat, mosquito vectors, mosquito ecology, larval development, cannibalism, *Aedes albopictus*

## Abstract

**Simple Summary:**

The consumption of conspecific individuals by cannibalism (i.e., the killing and eating of conspecific individuals) and necrophagy (i.e., feeding on dead individuals of the same species) has been documented in several insect species. Selective advantages have been proposed to explain the persistence of these behaviors in natural populations. In this paper, we tested the hypothesis that cannibalism and necrophagy lead to a significant resource loop within an insect cohort, allowing individual survival and development. With this aim, we performed laboratory and semi-field experiments using the tiger mosquito *Aedes albopictus*. Larval stages of this species develop in small aquatic habitats, such as tree holes and artificial containers. These latter are highly ephemeral habitats, where resources are often scarce and of poor quality (mainly leaf litter, wood, and vegetal detritus). We first estimated the relative rate of cannibalism and necrophagy. Then, we analyzed the effects of cannibalism and necrophagy on larval survival and adult yield. Consistent with our hypothesis, we found that conspecifics consumed about 50% of the initial cohort. Furthermore, conspecific consumption significantly increased the rate of adult emergence and larval survival, which supports that cannibalism and necrophagy can positively affect insect populations in temporary waters.

**Abstract:**

Temporary aquatic habitats are contingent on the allochthonous inputs of plant and animal detritus, whose quality and availability can significantly affect the species developing in these habitats. Although animal detritus (i.e., invertebrate carcasses) is a high-quality food, it is an unpredictable and variable resource. On the contrary, conspecific individuals (dead or alive) are a nutritionally high-quality food source that is always available. In this context, conspecifics consumption, by cannibalism or necrophagy, can be a good strategy to overcome nutrient limitation and allow individual maintenance and development. Here, we tested this hypothesis by using the tiger mosquito *Aedes albopictus*. By carrying out laboratory and semi-field experiments, we first estimated the relative rate of cannibalism and necrophagy, under different larval densities. Then, we analyzed the effects of cannibalism and necrophagy on larval survival and adult yield. Consistent with our hypothesis, we found that cannibalism and necrophagy occurred under all experimental conditions, and that conspecific consumption had positive effects on individual development, as it significantly increased the rate of adult emergence and larval survival. Interestingly, about 50% of the initial cohort was consumed by conspecifics, suggesting that cannibalism and necrophagy can drive an important resources loop in temporary aquatic habitats.

## 1. Introduction

Temporary waters are bodies of water mainly filled by rain, river overflow, or artificial flooding for agricultural purposes, where recurrent dry phases of varying duration occur [1]. They range from larger and more permanent water bodies (macro-habitats) to very small and highly ephemeral habitats, such as tree holes, phytotelmata, and man-made containers (micro-habitats) [1]. These latter are rapidly changing habitats, where the primary production is essentially absent, and the resource input is largely based on detritus of allochthonous sources, such as terrestrial leaf litter, wood and animal necromass (i.e., insect exuviae, invertebrate carcasses) [1,2]. This decaying organic matter is processed during decomposition, and nutrients are released into the water column, allowing the growth of the microbial community upon which protozoans and aquatic insects feed [3,4].

Empirical evidence has shown that the quantity and quality of available detritus can significantly affect the species living in temporary waters [1,3,5,6]. Animal detritus supports greater microorganism populations and productivity than leaf detritus, as it decomposes faster and releases more nutrients [4,7]. Furthermore, it lacks secondary plant compounds, such as tannins, that can negatively affect size, survival and growth rates of insects developing in temporary waters [8,9,10,11]. However, the availability of animal detritus is highly variable and fortuitous and can be an important constraint for species developing in these habitats. All organisms, indeed, need an energy and body-building supply for the maintenance and production of tissues [12,13]. In the environments, this supply is furnished by food. Once ingested, the short-lived organic molecules composing food are degraded by physiological processes and the contained energy and nutrients are used for self-maintenance and reproduction [12]. According to the stoichiometric approach, a balanced intake of nutrients is essential for individuals, and several evidences have shown that dietary mismatches can have deleterious effects on organisms, reducing their fitness [14,15]. Considering the low availability of high-quality food occurring in temporary waters, the possibility to obtain a balanced diet can be very challenging.

Here, we argue that the consumption of conspecific individuals, by cannibalism and necrophagy, can be a good strategy to avoid nutrient limitation in these habitats and obtain a near-perfect stoichiometric match between consumer and diet. Indeed, while detritus is fortuitous, conspecifics are always present and also, having a similar nutrient composition of consumer, a nutritionally much better food than vegetal detritus [6]. In this context, therefore, cannibalism or necrophagy, by reducing the stoichiometric mismatch between consumer and its food, can play a major role in supporting larval development and adult emergence.

In insect species, cannibalism has been frequently observed, and several significant ecological consequences have been highlighted. From a population perspective, cannibalism can regulate the population size, increase populations’ stability, and reduce the risk of extinction. It can also increase the resilience of populations to environmental stressors because cannibals and survivors are likely the more vigorous individuals within populations [16,17,18,19,20]. Finally, affecting individual dispersal, nutritional ability, and development time can also affect the colonization of new stressful environments [1,21,22]. At the individual level, cannibalism can be equally important because it can furnish nutritional advantages to the cannibal, with positive effects on development rate, survival and fertility [16,19]. Conspecific necrophagy, similarly to cannibalism, has been documented in several insects and selective advantages have been proposed to explain the persistence of this trait in natural populations [23,24]. In the ant *Formica polyctena* (Förster, 1850), conspecific necrophagy has been suggested as an adaptive strategy to satisfy nutritional requirements [25]. Likewise, in the boxelder bugs *Leptocoris trivittatus* (Say, 1825), conspecific necrophagy in overwintering individuals confers a survival advantage over bugs that do not feed on conspecifics, by providing additional nutritional resources [23]. To date, though cannibalism and necrophagy seem common behaviors, their combined contribution in overcoming the limitation of growth and development due to the availability of energy and body-building nutrients within an insect cohort is still poorly understood. In this paper, we used the tiger mosquito *Aedes albopictus* (Skuse, 1895) as a study-system to investigate this issue and test the hypothesis that cannibalism or necrophagy lead to a significant resource loop within an insect cohort, allowing individual survival and development.

Mosquitoes include more than 3500 species, and some of them, such as *Ae. albopictus*, are important disease vectors. The immature stages of mosquitoes develop in different aquatic sites, ranging from freshwater habitats to saltwater pools, where several inter- and intra-specific interactions can occur [16,19,26,27,28,29]. Events of conspecific consumptions have been documented in different mosquito genera, such as *Toxorhynchites*, *Armigeres*, *Aedes* and *Anopheles* and several factors have been shown to affect the extent of this behavior [30,31,32,33,34,35,36,37,38]. *Aedes albopictus*, among mosquitoes, represents one of the most invasive species in the recent decades. Originating from East Asia, it moved worldwide and established in all continents, occupying suburban and rural areas [39,40,41]. It is a major public health concern vectoring several arboviruses, such as dengue, chikungunya, Zika [42,43,44]. *Aedes albopictus* larvae develop in natural ephemeral water bodies, such as tree holes and man-made containers, such as tires, small water-holding containers, and cemetery vases [7,45]. In these habitats, the conditions are highly variable and the competition between individuals can be extremely high, which can affect survival and development [45]. Here, by using laboratory and semi-field experiments, we first estimated the relative rate of cannibalism and conspecific necrophagy, under different larval densities, to assess the magnitude of the resource loop. Then, we analyzed larval survival, the timing of adult emergence and adult yield among groups of larvae that consumed and did not consume conspecifics, to investigate the effects of cannibalism and conspecific necrophagy. If these processes play a major role, we expected to observe: (i) a higher number of adults in the *Ae. albopictus* cohorts where conspecific consumption occurred; (ii) the occurrence of a positive effect on larval development when conspecific consumption occurred than when it did not.

## 2. Materials and Methods

### 2.1. Mosquitoes

The *Aedes albopictus* mosquitoes used in this study were F1 generation derived from eggs collected by using ovitraps in the urban area of Rome city (41°54′04 lat., 12°31′26 long.) and raised to adults in laboratory. Larvae were reared in plastic trays (height = 5 cm, width = 30 cm and length = 19 cm) filled with 800 mL of distilled water and fed with cat food Friskies^®^ Adult (0.85 mg/larva of cat food daily). Eclosed adults were identified using the morphological keys of Schaffner et al. [46], kept in 40 cm cubic cages and fed with 10% sucrose solution changed daily. Females were blood-fed with fresh mechanically defibrinated bovine blood using a thermostatic apparatus; eggs were laid on paper towels partially immersed in cups containing water, and then, they were dried and stored at 27 °C until the experiments were performed. Eggs were hatched by the force-hatching technique to ensure uniform larval age. In particular, to stimulate the hatching, the eggs were put inside a 1.0-L closed container holding 0.75 L dechlorinated water, 0.25 g of Bacto nutrient broth, and 0.05 g of yeast. Rearing was performed in a climatic room at 27 ± 2 °C, 75 ± 10% relative humidity and an L:D 16:8 h photoperiod.

### 2.2. Experiment 1: The Consumption Rate of Conspecific Individuals

*Laboratory experiments*. We investigated the rate of conspecific consumption by cannibalism and necrophagy during larval development from L1 instar to adulthood in laboratory microcosms. Ten, fifteen and twenty-five L1 larvae (<24 h old) were placed into plastic containers (15 × 15 × 12 cm) filled with 400 mL of distilled water, leading to a density of 0.025, 0.04 and 0.06 larvae/mL, respectively [38]. These densities reproduced natural larval densities observed in mosquitoes, for example, in Italy, 0.0012–0.0895 larvae/mL were reported from different containers [47], and in USA, 0.0014–0.08 larvae/mL were observed [48]. Experiments were performed under the same food, temperature, humidity and light/dark conditions described above. For each density, five experimental containers were used, and the experiment was replicated three times. Distilled water was added as needed to maintain the initial volume.

Alive and dead larvae and pupae were counted daily at a fixed time (between 14:30 and 15:30 p.m.), stopping only when all larvae disappeared, were dead or emerged as adults. To assign the event of cannibalism and necrophagy, a practical definition was used. An event of cannibalism was assigned for each missing larva or pupa [37,38]. An event of necrophagy was considered when a dead larva or pupa that was observed one day, disappeared the following day. Twenty-five L1, L2, L3 and L4 dead larvae (killed through freezing at −80 °C) were placed into plastic trays as described above in absence of living larvae and used as control for possible larval decomposition and disappearance. Controls were performed in triplicate and missing larvae were counted daily during the following 72 h.

*Semi-field experiments*. The rate of conspecific consumption by cannibalism and necrophagy from L1 larvae to adulthood was assessed in semi-field microcosms. The experimental trials were located at an open green area inside the Department of Environmental Biology (Sapienza University, Rome) (41°53′45.92″ N, 12°31′2.19″ E), avoiding the access to unauthorized personnel. Plastic containers (15 × 15 × 12 cm) were filled with 400 mL of water and L1 larvae were added so as to obtain a density of 0.025, 0.04 and 0.06 larvae/mL. To provide nutrients, before the start of the experiments and the introduction of larvae, rearing water was infused for 3 days with yeast (0.5 g/L) and polyphite herbs (6 g/L) to allow microbial growth. Then, as in ephemeral micro-habitats necromass of invertebrate species can also be present, carcasses of *Drosophila suzukii* (Matsumura, 1931) killed by freezing were also added. The experiments started in late September, and we monitored the conditions of temperature with a digital datalogger (Hobo1, Onset Corp.), which sampled the environment once per hour. The mean temperature during this period was 17.25 °C (range 14.8–25.3 °C). To avoid colonization by insects, the containers were covered with netting having a mesh that allows gas exchange. Alive and dead larvae and pupae were counted daily at a fixed time (between 14:30 and 15:30 p.m.), stopping only when all larvae disappeared, were dead or emerged as adults. Cannibalism and necrophagy events were assigned as described above and all the experiments were performed in triplicate. Twenty-five L1, L2, L3 and L4 dead larvae (killed through freezing at −80 °C) were placed into plastic trays as described above in absence of living larvae and used as control for possible larval decomposition and disappearance.

*Data analysis*. The effect of density, developmental stage and their combination on the rate of cannibalism and necrophagy was investigated by Generalized Linear Models using a binomial error distribution and a logit link function. Analysis of Deviance was performed to investigate the effects of developmental stage (larval or pupal) and density and their combination on the cannibalism and necrophagy rate. The same approach was used to analyze the effect of density on the number of emerged adults. The fit of the model with the observed data was assessed using the Osius–Rojek test [49]. All analyses were performed using the software R version 3.6.2 [50].

### 2.3. Experiment 2: The Effects of Conspecific Consumption

We investigated the effects of cannibalism or necrophagy on larval development and adult yield. Two L1 larvae (<24 h old) were placed into 50 mL falcon tubes (15 × 15 × 12 cm) filled with 40 mL of distilled water. One-hundred-thirty tubes were used for a total of 260 larvae. Experiments were performed in a climatic room at 27 ± 2 °C, 75 ± 10% relative humidity and an L:D 16:8 h photoperiod and restricted food conditions (0.30 mg/larva of cat food daily).

Missing and dead larvae in each tube were counted daily at a fixed time (between 14:30 and 15:30 p.m.), stopping only when all larvae were dead or emerged as adults. As described above, an event of cannibalism was assigned when a missing larva or pupa was observed, and an event of necrophagy was assigned when a dead larva or pupa that was observed one day, disappeared the following day.

At the end of the experiments, the tubes were subdivided into three groups: “NC” group, where no larval consumption occurred; “CAN” group, where cannibalism occurred (one larva was cannibalized by the other larva); “NEC” group, where necrophagy occurred (one larva died and then was consumed by the other larva). For each group, we computed: (i) the proportion of larvae that reached the pupal and adult stages. They were computed as x/N, where x is the number of pupae or adults observed, and N is the total number of larvae for each group; (ii) the proportion of pupae that developed to adult stage; (iii) the mean time to adult emergence; (iv) the larval survival. Significant differences among groups were tested using 2 × 2 contingency tables and the Kruskal–Wallis test. Survival distributions of the three larval groups were computed using the Kaplan–Meier method [51], using the survminer R package [52]. In the CAN and NEC groups, we considered the larvae that consumed the other larva in each tube, and, in the NC group, we used the larva the survived longer in each tube. The differences between survival distributions were estimated using the Log-Rank Test. All analyses were performed using the software R version 3.6.2 [50].

## 3. Results

### 3.1. Experiment 1: The Consumption Rate of Conspecific Individuals

*Laboratory conditions*. Larvae, pupae and emerged adults were counted daily until all larvae were dead or emerged as adults. In the control tests, no larva disappeared, showing no larval decomposition during a 72-h time span. All events of necrophagy occurred during the 48 h after larval/pupal death.

**Figure 1:**
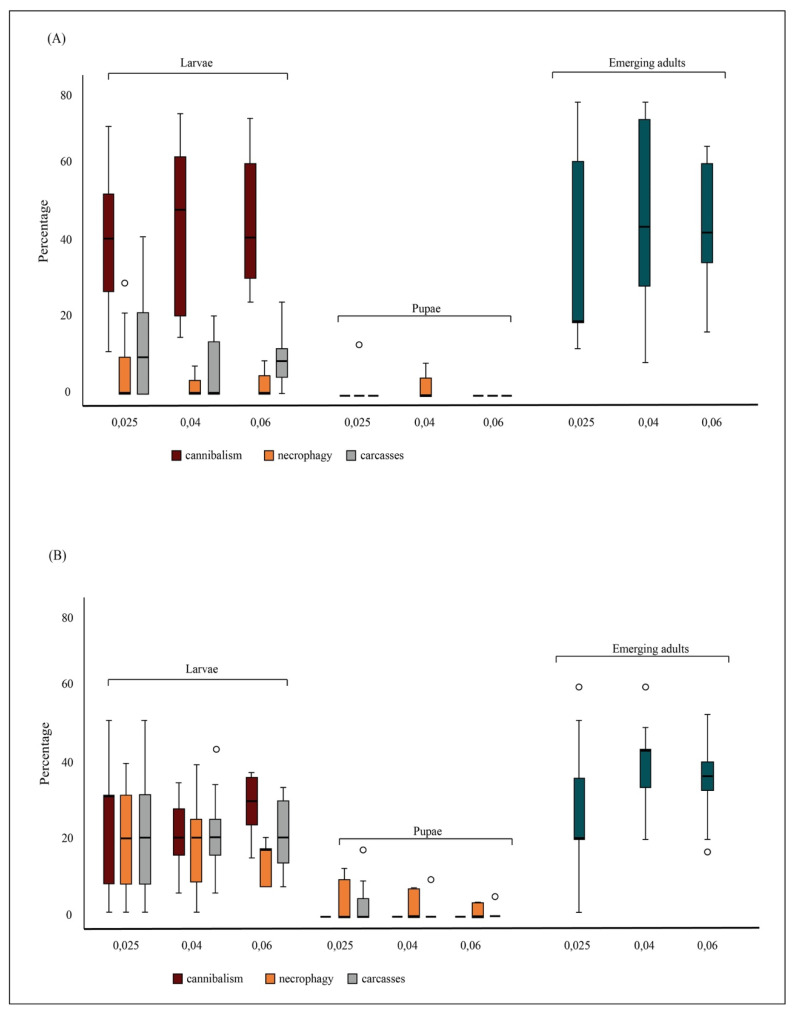
Rate of conspecifics’ consumption in *Aedes albopictus*. Panel (**A**) represents the percentage of cannibalism, conspecific necrophagy and remaining carcasses observed at each tested density and life-stage in laboratory experiments. Panel (**B**) represents the percentage of cannibalism, conspecific necrophagy and remaining carcasses observed at each tested density and life-stage in semi-field experiments. For each density, the percentage of emerged adults is also shown.

In the experimental trials, both cannibalism and necrophagy were observed among larvae, under all density conditions. A cannibalism event was observed and filmed using a smartphone camera during the daily count by an operator (Supplementary Materials Video S1). Larval cannibalism was 38.7% (±17.3), 42.2% (±21.9) and 43.8% (±15.0) at 0.025 and 0.04 and 0.06 larvae/mL densities, respectively. Larval necrophagy ranged from 7.0% (±8.1) to 12.0% (±14.7) at 0.04 and 0.025 larvae/mL densities, respectively (Figure 1A). Larval carcasses ranged from 1.9% (±3.2) to 6.7% (±9.8) at 0.04 and 0.025 larvae/mL densities, respectively (Figure 1A). Pupal cannibalism was not observed under any density conditions, while pupal necrophagy was observed only at 0.04 larvae/mL density (1.9% ± 3.2). Pupal carcasses were observed at the 0.025 larvae/mL density (4.0% ± 7.4) (Figure 1A). No significant differences between the model and the observed data were detected by the Osius–Rojek test (all *p*-values > 0.05). Cannibalism and necrophagy rates were not affected by density, while they were affected by the mosquito stage, with higher larval than pupal rate of cannibalism and necrophagy under all density conditions (Table 1, Figure 1A). Adult emergence rates were 38.7% (±26.7), 47.6% (±26.5) and 46.1% (±13.3) at 0.025, 0.04 and 0.06 larvae/mL densities, respectively. No significant effect of density on adult emergence rates was observed (Table 1).

*Semi-field conditions*. Missing larvae and pupae and emerged adults were counted daily until all larvae were dead or emerged as adults. In the control tests, no larva disappeared, showing no larval decomposition during a 72-h time span. In microcosms, both cannibalism and necrophagy were observed among larvae, under all density conditions. Larval cannibalism was 22.7% (±13.9), 20.9% (±7.5) and 27.2% (±7.3) at 0.025 and 0.04 and 0.06 larvae/mL densities, respectively. Larval necrophagy was 22.3% (±13.4), 21.3% (±9.5) and 21.1% (±8.5) at 0.025 and 0.04 and 0.06 larvae/mL densities, respectively (Figure 1B). Larval carcasses were 19.3% (±11.6), 17.8% (±10.9) and 13.3% (±4.5) at 0.025 and 0.04 and 0.06 larvae/mL densities, respectively (Figure 1B). Pupal cannibalism was not observed under any density conditions, while pupal necrophagy was observed at all densities, with values ranging from 0.5% (±1.7) at 0.04 and 3.6% (±6.8) at 0.025 larvae/mL densities (Figure 1B). Pupal carcasses were 5.3% (±7.4), 2.7% (±3.4) and 1.6% (±2.3) at 0.025 and 0.04 and 0.06 larvae/mL densities, respectively (Figure 1B).

No significant differences between the model and the observed data were detected by the Osius–Rojek test (all *p*-values > 0.05). Cannibalism and necrophagy rates were not affected by density, while they were affected by the mosquito developmental stage, with higher larval than pupal rate of cannibalism and necrophagy under all density conditions (Table 1, Figure 1B). Adult emergence rates were 26.7% (±15.8), 36.9% (±10.7) and 36.3% (±9.9) at 0.025, 0.04 and 0.06 larvae/mL densities, respectively. No significant effect of density on adult emergence rates was observed (Table 1).

### 3.2. Experiment 2: The Effects of Conspecific Consumption

In the experimental tubes, no larval consumption (neither cannibalism and necrophagy) was observed in 85 out of the 130 tubes, cannibalism was observed in 23 tubes and necrophagy in 22 tubes.

**Figure 2:**
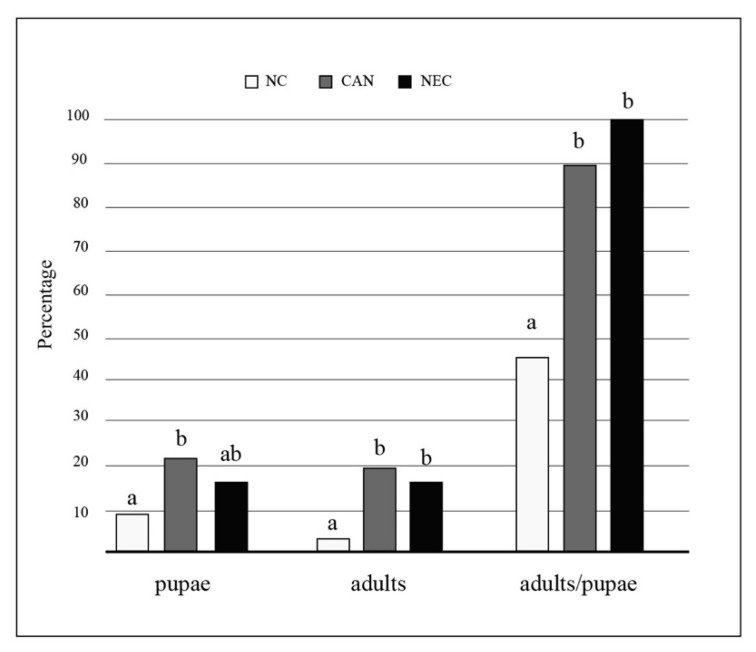
Effects of conspecifics’ consumption on larval development and adult yield in *Aedes albopictus*. The proportion of larvae that reached the pupal and adult stages and the proportion of pupae that developed to adult stage observed in each group are shown; “NC” group: no larval consumption occurred; “CAN” group: cannibalism occurred; “NEC” group: necrophagy occurred. Significant differences among groups were tested using 2 × 2 contingency tables. Equal letters mean no significant differences (chi-square tests, *p* > 0.05).

The proportion of larvae that reached the pupal and adult stages as well as the proportion of pupae that developed to adult stage were significantly different among the three groups, with higher values observed in the CAN and NEC groups than in the NC group (Figure 2). The time to adult emergence was not significantly different among the three groups (Kruskal–Wallis test, *p* = 0.084).

**Figure 3:**
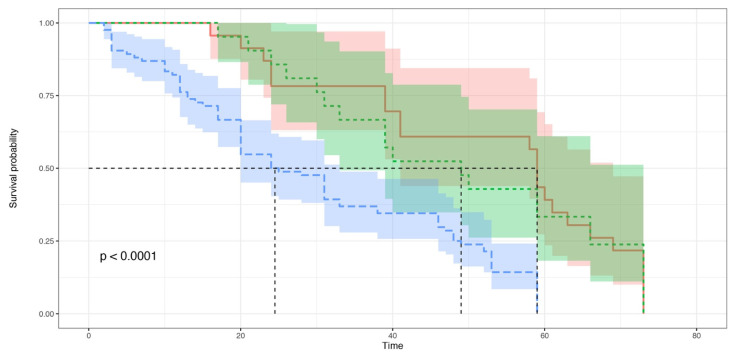
Kaplan–Meier survival curves. The *Aedes albopictus* larvae were grouped into three groups: the “NC” group (blue): no larval consumption occurred; “CAN” group (red): cannibalism occurred; “NEC” group (green): necrophagy occurred. The horizontal axis (x-axis) represents time (expressed in days), and the vertical axis (y-axis) shows the survival probability. At time zero, the survival probability is 1.0 (or 100% of the individuals are alive). The dotted lines show the median survival.

The Kaplan–Meier survival curves were built for the survival analysis of the *Ae. albopictus* larvae belonging to the NC, CAN and NEC groups (Figure 3). The observed survival distributions were significantly different between the three groups (Log-Rank score = 21.16, df = 2, *p* = 0.00017); between the NC and CAN groups (Log-Rank score = 18.76, df = 2, *p* = 1 × 10^−5^) and between the NC and NEC groups (Log-Rank score = 10.14, df = 2, *p* = 0.0062); no significant difference was observed between the CAN and NEC groups (Log-Rank score = 0.04, df = 1, *p* = 0.82).

## 4. Discussion

The availability of high-quality resources can be an important constraint for species living in ephemerous habitats. In this paper, by using laboratory and semi-field experiments with the mosquito *Ae. albopictus*, we showed that conspecific consumption, by cannibalism and necrophagy, can support larval development and adult emergence. In our first experiments, we showed that cannibalism and necrophagy occurred during development from L1 to adulthood at all tested densities, in both laboratory and semi-field microcosms. Even though the method here used to assign missing individuals could underestimate the relative contribution of necrophagy, because rapidly-consumed dead larvae (i.e., <24 h post-mortem) can be mistaken with cannibalistic events, our results showed that about 50% of the cohort biomass is consumed by conspecifics. In semi-field microcosms, a higher necrophagy rate was observed compared to laboratory conditions, which could be explained by the more variable temperature and food supply occurring in the field, that can increase mosquito mortality, and thus, the availability of necromass for the living larvae.

Under all tested densities, laboratory and semi-field microcosms concordantly showed a higher consumption of larvae than pupae. This difference was expected considering the mosquito life-cycle. Contrary to pupae that do not feed, mosquito larvae spend much time searching for food and, going through four instars, they have more opportunities to consume each other during development. Furthermore, the pupae are harder to consume because they swim more rapidly than larvae and, when fully formed, they have a sclerotized cuticle that is much thicker than the larval cuticle [26]. In accordance with this, Bara et al. [24] observed that *Ae. albopictus* larvae rapidly consumed larval detritus, while pupal detritus was consumed at a slower rate, and it was suggested that larvae are unable to chew through the sclerotized cuticle of mosquito pupae.

In addition, we found that neither cannibalism nor necrophagy were density-dependent. While, to the best of our knowledge, there are no studies that have investigated the relationship between conspecific necrophagy and larval density in mosquitoes, relative to cannibalism, some evidence showed that density can affect the rate of cannibalism, by increasing the competition for food or space, or by increasing the probability of encounter between the cannibal and the potential victims [20,31,32,36,38]. Although the density range here tested may not have been sufficiently high to detect an effect, in some studies, cannibalism was not associated with density even at high density, such as in the mosquitoes *Aedes triseriatus* (Say) [31] and *Armigeres subalbatus* (Coquillett) [33]. At an individual level, cannibalism has been suggested as an adaptive behavior by providing a high-nutrient dietary supplement that is beneficial for completing development [17,18,19,20]. The need for such a dietary supplement is independent of density, and is particularly important under extremely low nutrient conditions, such as in temporary waters. Thus, the conspecific consumption rate that we observed could be explained by the nutritional benefits of cannibalism and necrophagy. The results of our second experiment, where we investigated the benefits of conspecific consumption on larval development and adult yield, are consistent with this hypothesis.

Both cannibalism and necrophagy significantly increased the adult emergence rate and larval survival. The proportion of larvae that reached the pupal and adult stages, and the proportion of pupae that developed into adults were, indeed, significantly higher in the cannibalism and necrophagy groups than in the group where conspecific consumption did not occur. Furthermore, larval survival was significantly higher in the cannibalism and necrophagy groups. A similar effect of cannibalism or necrophagy was observed in other insect species, including mosquitoes. In *Ae. albopictus* and *Ae. aegypti* (Linneus, 1762), it was observed that the addition of *Drosophila melanogaster* (Meigen, 1830) carcasses produced more adults, with large additions producing the greatest survivorship [53]. In larvae of *D. melanogaster*, it was shown that an exclusively cannibalistic diet was enough for normal development from eggs to fertile adults [54]. Snyder et al. [55], who investigated the dietary benefits of cannibalism in larvae of the multicolored Asian lady beetle *Harmonia axyridis* (Pallas), found that cannibalism did not directly affect fitness, but increased larval survivorship. Likewise, in the pierid *Ascia monuste* (Linneus, 1764), cannibal caterpillars showed higher survival rate and weight than noncannibal ones [56]. In the Neotropical mosquito *Trichoprosopon digitatum* (Rondani, 1848), no measurable advantage of cannibalism was found in terms of emergence time or adult size, while larvae that consumed first instar larvae survived significantly longer than those which did not [57]. This increased survival is likely to provide an important advantage to mosquito larvae when they depend on the input of unpredictable food sources. Indeed, it may allow them to survive until the conditions become more favorable for their growth (for example, until leaves, fruit or animals fall into the water receptacle and promote microbial growth).

Taken together, our results showed that (i) both cannibalism and necrophagy occurred during larval development, with about 50% of the cohort biomass consumed by conspecifics; (ii) conspecific consumption has positive effects on individual development, as it significantly increases the rate of adult emergence and larval survival. Overall, these results support the hypothesis that the consumption of conspecific individuals can be a foraging strategy that allows larvae to overcome the effects of a variable supply of high-quality nutrients in temporary aquatic habitats. Future research on the population consequences of cannibalism and necrophagy in *Ae. albopictus* should focus on quantifying the effects of these interactions on fitness correlates of adults such as body size and fecundity.

The above findings can be interesting also from a vector control perspective. The British company Oxitec has proposed a “late acting lethality” strategy to control mosquitoes based on a dominant lethal genetic system inducing mortality in engineered individuals [58]. Modeling studies showed that killing individuals in later developmental stages, such as the pupal stage, can be more effective for population control than inducing an early-acting lethality, as in the Sterile Insect Technique approach. The advantage of this strategy would originate because larvae, remaining at this stage for a while before dying, experience a high level of intra-specific competition, which prevents population growth. However, a significant percentage of the initial biomass can be consumed (dead or alive) by other individuals, positively affecting larval development and adult yield, as suggested by our results. Therefore, if so, mosquitoes not carrying the lethal gene could consume the engineered individuals and survive, promoting the population increase. Future studies that explicitly consider conspecific consumption could shed light on this aspect.

## Figures and Tables

**Table 1:** Analysis of Deviance performed on cannibalism and necrophagy data from experiments performed under laboratory and semi-filed conditions. *** *p* < 0.001.

Variable	Df	Deviance Resid.	Df Resid.	Dev	Pr(>Chi)
**Laboratory conditions**					
**Cannibalism rate**					
Null			86	611.00	
stage	1	515.43	88	97.57	2 × 10^−16^ ***
Density	1	0.38	87	95.19	0.537
Stage × Density	1	0.00	86	95.19	0.999
**Necrophagy rate**					
Null			89	168.11	
stage	1	71.79	88	96.32	2 × 10^−16^ ***
Density	1	0.30	87	96.02	0.581
Stage × Density	1	1.42	86	94.60	0.233
**Adult yield**					
Null			44	137.40	
Density	1	0.930	43	136.47	0.335
**Semi-field conditions**					
**Cannibalism rate**					
Null			89	317.05	
stage	1	279.203	88	37.85	2 × 10^−16^ ***
Density	1	2.599	87	35.25	0.1070
Stage × Density	1	0.000	86	35.25	0.9999
**Necrophagy rate**					
Null			89	266.596	
stage	1	187.488	88	79.108	2 × 10^−16^ ***
Density	1	0.708	87	78.400	0.40008
Stage × Density	1	3.947	86	74.453	0.05051
**Adult yield**					
Null			44	51.545	
Density	1	2.334	43	49.212	0.1266

## Data Availability

Data can be found within the article and the Supplementary Materials.

## Supplementary Materials

The following are available online at https://www.mdpi.com/article/10.3390/insects12070657/s1, Video S1: Cannibalism in *Aedes albopictus* larvae. The movie shows a cannibalism event in *Aedes albopictus*. The event was observed and filmed using a smartphone camera during the daily count by an operator.
